# Lifestyle and eating habits before and during COVID-19 quarantine in Brazil

**DOI:** 10.1017/S136898002100255X

**Published:** 2021-06-10

**Authors:** Tamires CM Souza, Lívya A Oliveira, Marina M Daniel, Lívia G Ferreira, Ceres M Della Lucia, Juliana C Liboredo, Lucilene R Anastácio

**Affiliations:** 1 Department of Food Science, Faculty of Pharmacy, Universidade Federal de Minas Gerais, Av. Pres. Antônio Carlos, 6627, Pampulha, Belo Horizonte, MG 31270-901, Brazil; 2 Department of Nutrition and Health, Universidade Federal de Viçosa, Viçosa, MG, Brazil; 3 Department of Nutrition, Universidade Federal de Lavras, Lavras, MG, Brazil; 4 Department of Food, Universidade Federal de Ouro Preto, Ouro Preto, MG, Brazil

**Keywords:** Coronavirus, SARS-CoV-2, Food choices, Alcohol use, Smoking, Lockdown

## Abstract

**Objective:**

To assess changes in daily habits, food choices and lifestyle of adult Brazilians before and during the COVID-19 pandemic.

**Design:**

This observational study was carried out with Brazilian adults through an online questionnaire 5 months after the social distance measures implementation. The McNemar, McNemar–Bowker and Wilcoxon tests were used to investigate differences before and during the COVID pandemic period, adopting the statistical significance of *P* < 0·05.

**Setting:**

Brazil.

**Participants:**

Totally, 1368 volunteers aged 18+ years.

**Results:**

The volunteers reported a lower frequency of breakfast, morning and lunch snacks (*P* < 0·05) and a higher frequency of evening snacks and other meal categories during the pandemic period *(P* < 0·05). The results showed an increase in the consumption of bakery products, instant meals and fast food, while the consumption of vegetables and fruits decreased (*P* < 0·005). There was a significant increase in the frequency of consumption of alcoholic beverages (*P* < 0·001), but a reduction in the dose (*P* < 0·001), increased frequency of smoking (*P* = 0·007), an increase in sleep and screen time in hours and decrease in physical activity (*P* < 0·001).

**Conclusions:**

It was possible to observe an increase in screen time, hours of sleep, smoking and drinking frequency. On the other hand, there was a reduction in the dose of alcoholic beverages but also in the practice of physical activity. Eating habits also changed, reducing the performance of daytime meals and increasing the performance of nighttime meals. The frequency of consumption of instant meals and fast food has increased, while consumption of fruits and vegetables has decreased.

## Graphical abstract

**Figure:**
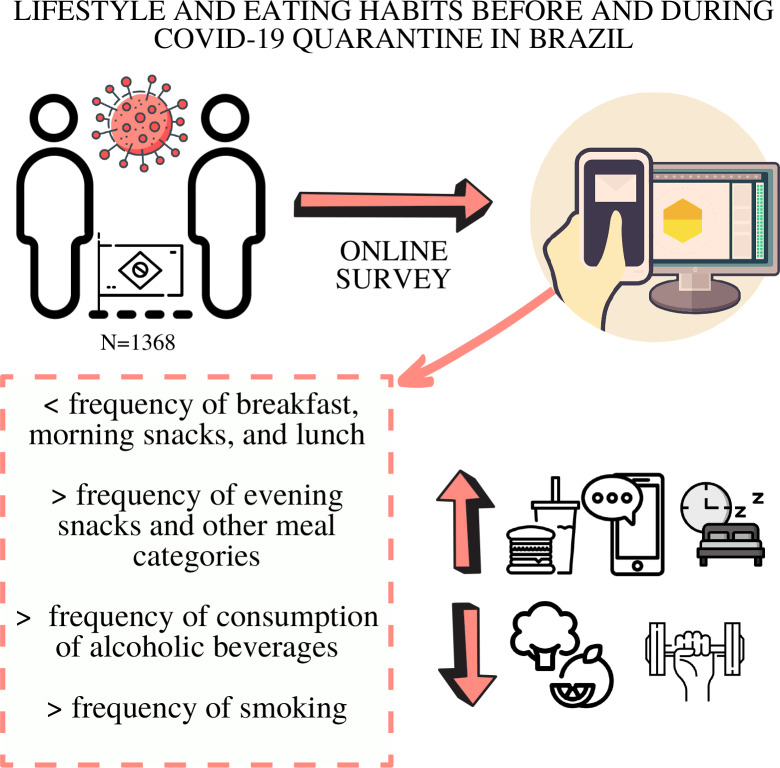

At the end of December 2019, the Chinese authorities informed on the cluster of lung infections due to unknown aetiological factors, later identified as a consequence of transmission of the new coronavirus (SARS-CoV-2)^(1)^. SARS-CoV-2 is part of a family of viruses common in numerous animal species, and its infection culminates in an acute respiratory disease named COVID-19, which can be asymptomatic or take on a serious clinical condition^(1)^.

The WHO declared a Public Health Emergency of International Importance in January 2020. According to the terms of International Health Regulations, this situation represents the highest level of alert provided by WHO. This alert prompted countries to take different security measures to minimise transmission, but despite initial efforts, in March 2020, COVID-19 was declared a pandemic by WHO^(2)^. At the beginning of May, more than 150 million cases had already been confirmed, and the number of deaths by COVID-19 was over 3·2 million, with Brazil being one of the countries with the most dramatic situation in the world: more than 15 million cases, exceeding 420 thousand deaths^(3)^.

The transmission of the SARS-CoV-2 occurs mainly via respiratory droplets quickly, through close contact with contaminated people, and one of the main ways found to stop the spread of the new coronavirus was to establish physical and social distancing measures, in addition to other safety measures, such as washing hands frequently, sanitise places of common use, wearing protective masks and interrupt the offer of services not classified as essential^(2)^. Among all, quarantine promoted physical distancing, considered the most effective way to prevent infection by SARS-CoV-2.

Studies carried out in different populations have already identified that the security measures adopted to face the new coronavirus, as the lockdown and home quarantine, promoted a lot of changes^(4)^ and interfered in habits and lifestyle, including increased alcohol consumption and smoking frequency^(5)^, reduced physical activity^(6)^ and changes in dietary patterns and food purchases^(7–9)^. However, studies that evaluate these changes in the Brazilian population are incipient, but already show small changes in the eating habits of adolescents^(10)^, worsening in the quality of life of adults^(11)^ and a possible trend of worse eating patterns in underdeveloped regions of the country^(12)^.

Thus, further research on the behaviours before and during lockdown measures adopted in Brazil is necessary, so that it is possible to carry out necessary interventions to minimise harmful effects in terms of health and quality of life in general. Therefore, the aim of this research was to assess changes in daily habits, food choices and lifestyle of adult Brazilians before and during the COVID-19 pandemic.

## Methods

### Study design and participants

**Fig. 1:**
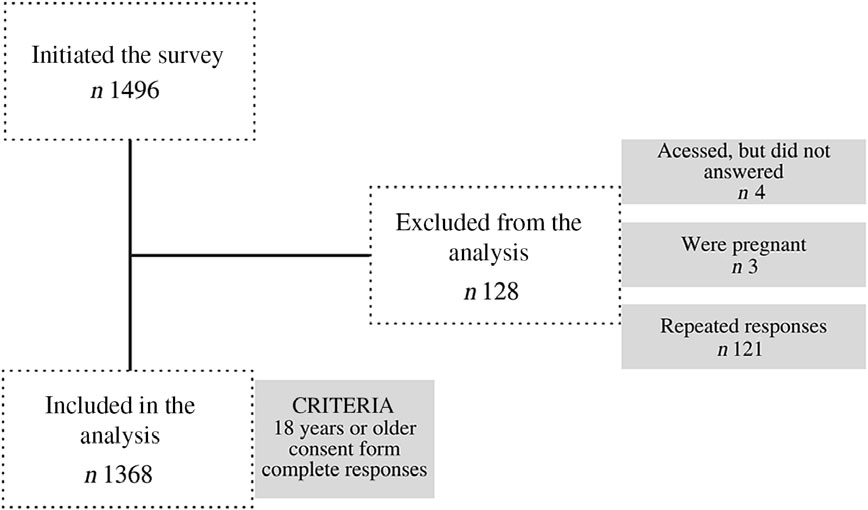
Study recruitment

This is an observational study conducted with the Brazilian population, in which data related to daily habits (variables related to time and form of work and time of sleep), lifestyle (screen time, smoking and drinking habits and physical activity) and eating habits were collected during the COVID-19 pandemic. The survey was conducted with Brazilian volunteers, 18 years old or older, who agreed to participate and answered an online questionnaire. Pregnant women were excluded from the sample (Fig. 1). The study was conducted according to the Declaration of Helsinki^(13)^ and was approved by the institutional Research Ethics Committee.

### Data collection

The online questionnaire was created on the Google Forms® search management application and was enabled for responses during 27 d from August to September 2020 – approximately 5 months after the implementation of quarantine. In Brazil, the lockdown measures implemented during that period included (1) suspension of nonessential activities (closing of restaurants, bars, shopping malls and gyms); (2) suspension of schools and universities’ activities and implementation of emergency remote education and (3) an incentive to adhere to social and physical distance measures, among other issues addressed in Federal Law No. 13 979, 6 February 2020^(14)^. For this study, we defined the pre-pandemic period as before January 2020. Information about the survey and the link to access the questionnaire were publicised on the university’s websites and social media. The volunteers could access the questionnaire through any device that had access to the internet, and the response time was around 15 min. When accessing the link, volunteers were directed to the consent form and had the option of consenting to participate or not. Access to the form was given to those who accepted it and, after providing their contact information, the volunteers received by email a copy of the properly signed consent form.

All responses were documented anonymously and saved only when the volunteers selected the ‘Submit’ button. Thus, participants were able to stop their participation in the study at any stage before the submission of the answers. The complete survey was sent to the final database and downloaded as a Microsoft Excel archive.

### Variables

The collected variables were divided into three groups of questions. The first group consisted of personal and daily habits, such as age, gender, educational level, personal income, composition of the household, the practice of quarantine, current occupation and perception about the working time during COVID-19 pandemic. The subsequent groups of questions consisted of collecting self-reported lifestyle and eating habits variables before and during the pandemic.

Concerning lifestyle, questions about frequency and dose of alcoholic beverage consumption, smoking, physical activity and sleep time were collected. The habit of drinking alcoholic beverages was investigated using frequency data (nondrinkers: rarely, once a week, 2–3 times/week, 4–6 times/ week and every day) and ingested dose. The smoking habit was investigated through categories divided into units/d. Screen time (smartphones, computer, tablet and TV) before and during the pandemic was assessed by hours, distributed as follows: <4 h/d, 5–8 h/d, 9–12 h/d, 13–16 h/d and >16 h/d. The frequency of physical activity was assessed based on six categories: 0 min/week, <90 min/week, 91–150 min/week, 151–210 min/week, 211–270 min/week and >271 min/week. Self-reported data were used to assess bedtime and wake up, and the difference between times before and during the pandemic was calculated.

To investigate eating habits, questions related to the number of meals were carried out, and an FFQ was applied. For the frequency questionnaire, the categories were (1) fresh fruits and legumes (beans, soybeans, lentils and chickpeas); (2) cereals (rice, corn and oats); (3) bakery products, meat, milk and dairy; (4) vegetables (not considering potatoes, manioc/cassava and yams); (5) instant meals and snacks (noodles, packaged snacks or crackers); (6) sweetened drinks (soda, canned or powdered juice, canned coconut water, guarana/blackcurrant syrup and sugared fruit juice); (7) candies (chocolates, pies, gum, caramel and gelatin); (8) hamburgers and canned products (ham, bologna, salami and sausage) and (9) fast food (pizza and sandwiches). For each food category, participants had the options of the frequency of consumption: never, rarely, once a week, 2 to 3 time/week, 4 to 6 time/week and once a day and more than once a day.

### Statistical analysis

Data were evaluated using the Statistical Package for Social Science version 22.0 (SPSS Inc.). The Kolmogorov–Smirnov test was applied, and all variables showed a non-parametric distribution. Thus, data were presented in tables and figures, with frequency values (absolute number and percentage), as well as median and interquartile range. McNemar and McNemar–Bowker tests (Bonferroni adjusted) were used to investigate the differences in categorical variables before and during the COVID-19 pandemic period. Wilcoxon test was applied for the comparisons between numerical variables. Lifestyle habits were categorised according to the quartiles that represented the worst outcome, being: frequency of alcohol consumption equal to or greater than once a week (last quartile); dose of alcoholic beverages equal to or greater than 2·5 (last quartile); screen time equal to or greater than 10·5 h (last quartile); physical activity equal to zero minutes a week (first quartile) and hours of sleep equal to or less than 7 h (first quartile). Smoking habits were analysed among those that did not smoke and the ones that did. Based on this categorization, univariate and multivariate logistic regression models were obtained by the enter method. All the covariates with *P* < 0·20 on univariable analysis (see online supplemental data) were entered in the initial model. The fit of the models was tested by the Hosmer and Lemeshow test (*P* > 0·05). The statistical significance was determined as *P* < 0·05.

## Results

A total of 1496 participants answered the questionnaire, and after data validation, 1368 respondents were included in the study. The median age of participants was 31·0 (24·0–39·0) years, and the sample was composed mainly of females (80·0 %), people with a complete degree (36·2 %), living with their parents (38·3 %) and practicing quarantine totally (57·2 %) or partially (39·8 %) (Table 1). Volunteers from different Brazilian regions attended the study, but most respondents (89·6 %) reside in the southeast region.

**Table 1:** Participants’ general characteristics (*n* 1368)

Variable	Median (Q1 – Q3) frequencies (%)	*n*
Gender		
Female	80·0	1094
Male	19·7	269
No answer	0·3	5
Age		
Years	31·0	24·0–39·0
Education level		
Complete primary education	0·1	2
Incomplete high school	0·3	5
Complete high school	5·1	70
Incomplete graduation	28·4	389
Complete graduation	19·4	266
Incomplete postgraduate studies	10·4	141
Complete postgraduate studies	36·3	495
Per capita income		
US$	347·46	78·37–352·68
Composition of people living in the same household during COVID-19 pandemic		
Alone	8·0	110
With friends, brothers and other	11·1	152
With husband/wife	17·4	238
With husband/wife and children/with children	25·2	344
With parents	38·3	524
Social isolation during COVID-19 pandemic		
Total	57·2	783
Partial	39·8	544
No	3·0	41
Occupational situation during COVID-19 pandemic		
Unemployed	7·3	100
Retired	3·3	45
Work/study remotely full time	40·6	555
Work/study remotely part-time	30·1	412
Work/study unchanged, not remotely	11·0	150
Other	7·7	106
Perception of working time during the pandemic (including housework)		
Increased	65·9	902
Decreased	12·8	175
Remained the same	21·3	291

There were significant differences between the periods before and during the COVID-19 pandemic, considering the frequency of alcohol consumption, smoking habits, screen time, physical activity and sleeping time (Table 2). These results indicated an increase in the frequency of consumption of alcoholic beverages, but a reduction in the dose, an increase in the frequency of smoking, but no significant difference in the number of cigarettes smoked per day, an increase in sleep and screen time in hours and a reduction in physical activity in terms of frequency and weekly minutes.

**Table 2:** Lifestyle habits before and during the COVID-19 pandemic

Variable		Before		During	
Frequency of alcoholic beverage		*n* 1323		*n* 1323	
Median (Q1 – Q3)	Frequency/week	0·5	0·00–1·00	0·5	0·00–1·00
*P* value		0·022 (Wilcoxon test)			
Frequency % (*n*)	Nondrinkers	26·3 %	348	29·8 %	395
	Rarely	27·6 %	366	29·2 %	386
	Once a week	23·1 %	305	16·9 %	224
	2–3 times/week	20·9 %	277	18·4 %	243
	4–6 times/week	1·6 %	22	4·9 %	65
	Every day	0·5 %	5	0·8 %	10
*P* value		<0·0001(McNemar–Bowker test: 96·38)			
Dose of alcoholic beverage		*n* 1354		*n* 1354	
Median (Q1 – Q3)	Dose per occasion	2·5	0·00–2·5	1·0	0·00–2·5
*P* value		<0·0001(Wilcoxon test)			
Frequency % (*n*)	None	26·7 %	362	30·2 %	408
	One dose	21·3 %	288	25·9 %	351
	2–3 doses	22·2 %	395	26·3 %	356
	4–5 doses	11·3 %	154	9·3 %	126
	≥6 doses	11·5 %	155	8·3 %	113
*P* value		<0·0001(McNemar–Bowker test: 77·551)			
Cigarette		*n* 1324		*n* 1324	
Median (Q1 – Q3)	Units/d	0·00	0·00–0·00	0·00	0·00–0·00
*P* value		0·227(Wilcoxon test)			
Frequency % (*n*)	Nonsmokers	94·8 %	1255	95·1 %	1259
	<10 cigarettes/d	4·2 %	57	3·1 %	41
	11–20 cigarettes/d	0·8 %	10	1·2 %	16
	21–30 cigarettes/d	0·1 %	1	0·4 %	5
	≥31 cigarettes/d	0·1 %	1	0·2 %	3
*P* value		0·007 (McNemar–Bowker test: 19·364)			
Screen time		*n* 1311		*n* 1311	
Median (Q1 – Q3)	h/d	6·50	3·00–6·50	10·00	6·50–10·50
*P* value		<0·0001(Wilcoxon test)			
Frequency % (*n*)	<4 h/d	43·6 %	572	13·6 %	177
	5–8 h/d	37·9 %	496	30·9 %	406
	9–12 h/d	15·4 %	202	6·5 %	86
	13–16 h/d	2·5 %	33	42·1 %	551
	>16 h/d	0·6 %	8	6·9 %	91
*P* value		<0·0001(McNemar–Bowker test: 804·910)			
Physical activity		*n* 1347		*n* 1347	
Median (Q1 – Q3)	Min/week	120·00	0·00–180·00	80·00	0·00–120·00
*P* value		<0·0001(Wilcoxon test)			
Frequency % (*n*)	Sedentary	27·4 %	369	38·7 %	521
	<90 min/week	16·4 %	221	21·8 %	294
	91–150 min/week	20·9 %	282	16·2 %	218
	161–210 min/week	14·6 %	197	10·9 %	147
	211–270 min/week	8·5 %	113	5·7 %	77
	>270 min/week	12·2 %	165	6·7 %	90
*P* value		<0·0001(McNemar–Bowker test: 137·508)			
Sleep time		*n* 1352		*n* 1352	
Median (Q1 – Q3)	Hours	8:00	7:00–8:30	8:00	7:00–9:00
	Time to sleep (h)	10:00 PM	11:00 PM–4:00 AM	9:30 PM	11:00 PM–1:00 AM
	Time to wake up (h)	6:30 AM	6:00 AM–9:00 AM	7:30 AM	6:50 AM–9:00 AM
*P* value		<0·0001(Wilcoxon test)			

The number of volunteers differed between variables since not all people answered questions before and after.

Table 3 contains all factors independently associated with: consumption of alcoholic beverages equivalent to once a week or more and more than 2·5 doses or more per occasion, smoking habit, screen time per day of 10·5 h or more, do not practice any time of physical activity and sleep 7 h or less per night.

**Table 3:** Independent factors associated with the lifestyle habits during the pandemic period in Brazil by multiple logistic regression analysis

	OR	95 % CI	*P* value
Frequency of alcohol consumption* (≥ once a week – last quartile)			
Variables (64·0 % of prediction; Hosmer and Lemeshow test = 0·246)			
†Per capita income (R$)	1·000	1·000, 1·000	0·001
Living with children	1·521	1·159, 1·996	0·002
Educational level (post-graduate)	1·599	1·245, 2·053	<0·001
Smoking during pandemic (cigarettes/d)	1·077	1·035, 1·120	<0·001
Frequency of instant meals and snacks consumption	0·949	0·903, 0·998	0·041
Physical activity during pandemic (min/week)	1·002	1·000, 1·003	0·022
Working or studying without alterations	2·004	1·399, 2·870	<0·001
Constant	0·317		<0·001
Dose of alcoholic beverage consumption* (≥ 2·5 doses per occasion – last quartile)			
Variables (62·6 % of prediction; Hosmer and Lemeshow test: 0·200)			
Gender (male)	1·392	1·050, 1·846	0·022
Living with parents	0·765	0·606, 0·967	0·025
Working or studying without alterations	1·820	1·274, 2·602	0·001
Smoking during pandemic (cigarettes/d)	1·105	1·055, 1·157	<0·001
Physical activity during pandemic (min/week)	1·003	1·001, 1·004	<0·001
Frequency of fresh fruits consumption	0·953	0·923, 0·985	0·005
Constant	1·037		0·834
Smoking habit (yes)			
Variables (92·2 % of prediction; Hosmer and Lemeshow test: 0·347)			
Age (years)	1·076	1·057, 1·096	<0·001
‡Per capita income (R$)	1·000	1·000, 1·000	0·006
Educational level (post-graduate)	0·563	0·348, 0·909	0·019
Dose of alcoholic beverage during pandemic (dose per occasion)	1·328	1·194, 1·478	<0·001
Daily breakfast during pandemic	0·256	0·156, 0·418	<0·001
Daily afternoon snack during pandemic	0·520	0·323, 0·837	0·007
Frequency of fresh fruits consumption (times/week)	0·933	0·875, 0·995	0·033
Frequency of sweetened drinks consumption (times/week)	1·112	1·048, 1·181	<0·001
Constant	0·029		<0·001
Screen time (≥ 10·5 h/d – last quartile)			
Variables (64·7 % of prediction; Hosmer and Lemeshow test: 0·264)			
Age (years)	0·965	0·955, 0·976	<0·001
Remotely full/part-time work or study	1·953	1·427, 2·673	<0·001
Working or studying without alterations	0·553	0·345, 0·888	0·014
Increase in time spent at work (including household chores)	0·552	0·430, 0·707	<0·001
Sleep time during pandemic (h/d)	0·859	0·785, 0·939	0·001
Physical activity during pandemic (min/week)	0·998	0·997, 1·000	0·008
Daily breakfast during pandemic	0·596	0·429, 0·829	0·002
Constant	15·294		<0·001
Physical activity (0 min/week – first quartile)			
Variables (67·6 % of prediction; Hosmer and Lemeshow test: 0·175)			
Gender (female)	1·615	1·181, 2·209	0·003
§Per capita income (R$)	1·000	1·000, 1·000	0·038
Living with children	1·478	1·128, 1·937	0·005
Remotely full/part-time work or study	0·610	0·471, 0·790	<0·001
Dose of alcoholic beverage during pandemic (dose per occasion)	0·856	0·800, 0·915	<0·001
Sleep time during pandemic (h/d)	0·881	0·805, 0·964	0·006
Daily afternoon snack during pandemic	0·632	0·465, 0·859	0·003
Frequency of bakery products consumption (times/week)	1·104	1·060, 1·150	<0·001
Frequency of fresh fruits consumption (times/week)	0·858	0·827, 0·889	<0·001
Frequency of meat consumption (times/week)	1·053	1·011, 1·096	0·013
Frequency of sweetened drinks consumption (times/week)	1·043	1·005, 1·082	0·027
Constant	2·527		0·039
Sleep time (≤7 h/d – first quartile)			
Variables (74·0 % of prediction; Hosmer and Lemeshow test: 0·278)			
Gender (male)	1·632	1·195, 2·227	0·002
Living with children	1·433	1·056, 1·944	0·021
Education level (graduation)	1·366	1·043, 1·790	0·024
Increase in time spent at work (including household chores)	0·509	0·378, 0·684	<0·001
Screen time during pandemic (h/d)	1·056	1·022, 1·090	0·001
Frequency of alcoholic beverage during pandemic (times/week)	0·900	0·814, 0·996	0·041
Daily afternoon snack during pandemic	0·591	0·433, 0·806	0·001
Daily evening snack during pandemic	1·505	1·143, 1·982	0·004
Constant	0·383		<0·001

* The frequency and dose of alcoholic beverages are highly correlated habits in the evaluated population (*r* = 0.806; *P* < 0.001), and, therefore, they were causing multicollinearity and interfering in the adjustments of their respective models. Therefore, the dose of alcoholic beverages was not included as a predictor of frequency of alcoholic beverage and vice versa.

† OR = 1.000095; IC = 1.000041, 1.000149.

‡ OR = 0.999819; IC = 0.999690, 0.999949.

§ OR = 0.999942; IC = 0.999886, 0.999997.

**Fig. 2:**
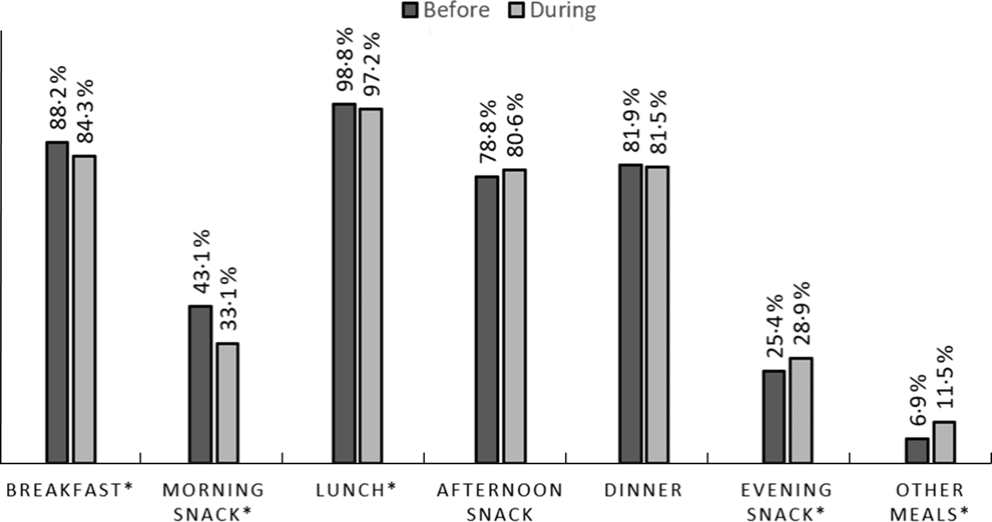
Comparisons between meals made by participants before and during the COVID-19 pandemic (*n* 1368). *McNemar test, respectively: *P* < 0·001; *P* < 0·001; *P* = 0·002; *P*= 0·003; *P* < 0·001

Consumption of meals changed significantly between the previous period and during the period of the pandemic, except dinner and afternoon snack (Fig. 2). The volunteers reported a lower frequency of breakfast, morning snack and lunch and a higher frequency of evening snack and other meals categories during the pandemic period.

**Fig. 3:**
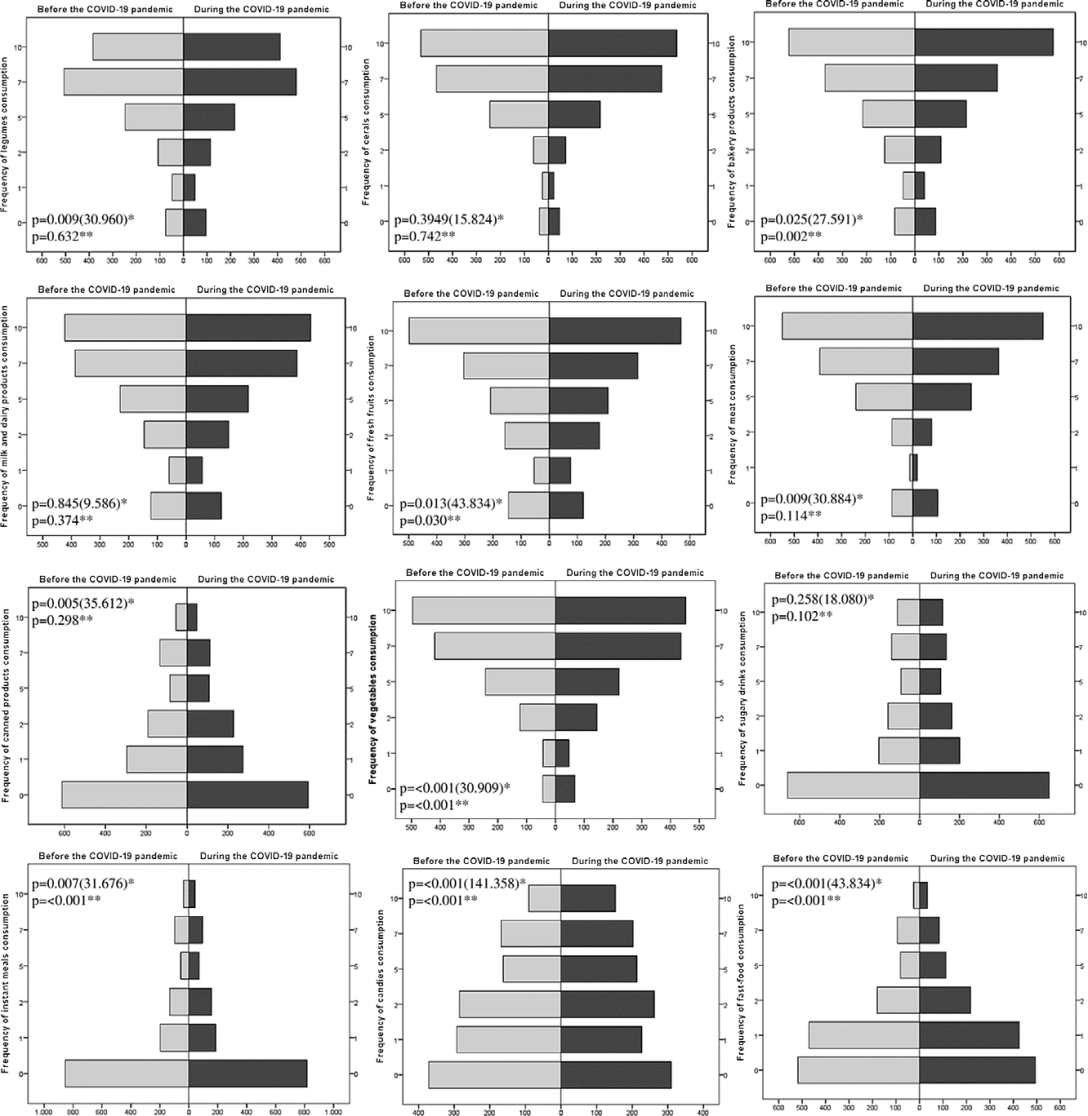
Frequency of food consumption before and during the COVID-19 pandemic (*n* 1368). *McNemar–Bowker Test. **Wilcoxon

Significant differences were observed between frequency of food consumption before and during COVID-19 pandemic, such as fresh fruits, legumes, bakery products, meat, vegetables, instant meals and snacks, candies, canned products and fast food (Fig. 3). The results of this study showed a significant increase in the consumption of bakery products, instant meals and fast food, while the consumption of vegetables and fruits decreased.

## Discussion

This study was dedicated to investigate the changes in daily habits, food choices and lifestyle of adult Brazilians before and during the COVID-19 pandemic. Our main results demonstrated that there was an increase in the screen time, in the hours of sleep, in the habit of smoking and in the frequency of alcoholic beverage ingestion. In addition, eating habits have also changed, and it was possible to observe a significant increase in the consumption of bakery products, instant meals and fast food, while the consumption of vegetables and fruits decreased. Also, the quartiles that represent the worst outcomes of each lifestyle habits evaluated were associated with several factors, including age, gender, per capita income, family composition, arrangement of work adopted during the pandemic period, eating habits, among other variables of lifestyle.

Physical and social distancing is one of the main security measures to combat the spread of the new coronavirus and is highly recommended by health authorities^(2)^. However, studies carried out with populations in other countries have shown that such measures can drastically affect life habits^(5,7–9)^. Our results demonstrate that only 3·0 % of our sample reported not to be fulfilling physical and social distancing at the study time. Therefore, we are confident that the data allowed us to observe the outcomes of interest. The study by Malta *et al.* ^(11)^ also carried out with the Brazilian population also reported a similar result, in which <2 % of the participants were not in quarantine. Our sample was composed mainly of women, which has become common in research investigating eating habits during the pandemic. In other studies carried out in different countries and populations, with people in different clinical conditions and ages, women have represented more than half of the volunteers^(5,8,15–18)^.

The frequency of alcoholic beverage intake has increased significantly during the pandemic in our sample. The increase in drinking frequency can be associated with an attempt to combat stress, boredom and possible negative emotions resulting from physical and social isolation^(19)^. Despite the increased frequency of alcoholic beverages, the dose of consumption has significantly decreased. The decrease in the alcohol consumption/occasion differs from that of most studies that assess lifestyle and COVID-19 have consistently demonstrated^(5,20,21)^. Along with the ban on the operation of bars and the holding of parties in Brazil, one of the possible explanations for the occurrence of these divergences is the fact that our sample is composed mainly of women. Although alcohol consumption has been growing significantly among women in the last decade, men still have a higher prevalence of excessive alcohol consumption^(22)^. The consumption of 2·5 doses or more of alcoholic beverages per occasion was also associated with the male gender in the present study (OR = 1·392). Also, the motivations for drinking alcohol may differ according to gender: men are more likely to drink when exposed to stress, while women prefer to drink in relaxation and entertainment situations^(22)^. In addition to our sample being composed mainly of women, more than a quarter of the interviewees reported living at home with children. Recent research has shown that women avoid consuming excessive doses of alcoholic beverages in this family composition, while men do not change this specific habit^(23)^. Interestingly, our data showed that living with children was a predictive factor for the consumption of alcoholic beverages once a week or more in our sample (OR = 1·521). However, living with parents was inversely associated with consuming, per occasion, 2·5 doses of alcohol or more (OR = 0·765).

Although the number of nonsmokers in our sample exceeds 90 %, our results showed that during the pandemic, the number of nonsmokers decreased. While the number of cigarettes/d increased in the categories of eleven units or more, despite the recommendations of the health authorities, who issued a warning that smoking is associated with an increase in the severity of the disease and death in hospitalised COVID-19 patients and advised that all support should be given to encourage the interruption of this habit^(24)^. Researchers have already shown that one of the reported reasons for smoking in unpleasant situations is that cigarettes seem to cause a momentary feeling of relief^(25,26)^. Other studies also observed an increase in the number of cigarettes during the pandemic period^(27,28)^. However, an Italian survey observed a decrease in smoking habits^(8)^. We hypothesised that due to the atypical content of the present moment, individuals who may have previously dropped the addiction may have faced the need to resume the use of cigarettes during the lockdown, and this habit was associated with the consumption of 2·5 doses or more of alcoholic beverages per occasion (OR = 1·328) and inversely associated having intermediate meals like morning snack (OR = 0·256) and afternoon snack (0·520), in addition to consuming fresh fruits more often (OR = 0·933) and sweetened drinks less often (OR = 1·112). Previous data reported that people who quit addictions are more prone to relapses and fluctuations in atypical and high-pressure periods^(29,30)^.

Another change observed was the increase in screen time, including television, computers, tablets and cell phones. More than half of our respondents reported an increase in working time during the pandemic – including housework. Most of the studied population reported being working/studying remotely full or part-time, and this reflects directly on screen time since people were led to adapt to a way of working called ‘intelligent’, in which the obligations are fulfilled remotely and, for the most part, online^(29)^. Besides working or study remotely (OR = 1·953), other factors were inversely associated with 10·5 h or more of screen time during the pandemic, like being older (OR = 0·965), working or studying without changes (OR = 0·553), increased time spent on work (including household chores) (OR = 0·552) and practicing physical exercise (OR = 0·998).

Undoubtedly, the use of devices during quarantine is an important tool for communication, as they can act as facilitators and can alleviate moments of loneliness. However, in some populations, when in excess, this behaviour negatively interfered in food choices, being associated with worse food choices, including higher consumption of ultra-processed foods^(31)^ and high consumption of snacks, fried foods and sweets^(32)^. Unfortunately, the increase in screen time has been a reality in other populations during the pandemic, having already been demonstrated in Canadians^(33)^ and Iranians^(34)^ and were related to the increase in sedentary lifestyle.

The findings related to the reduction in the practice of physical activity in the present study was already expected, and they are in line with current research. Many studies carried out during pandemic found changes in behaviours related to physical activity, such as 12 % increased sitting time among individuals in Italy^(35)^; 78 % reduction in the time of physical exercise of the Iranian population^(36)^, 79 % among Brazilians^(37)^ and more than 60 % in an analysis carried out in fourteen countries, compared with the period before the pandemic^(38)^. These changes are justified by the difficulty of exercising since, among security measures, gyms, training and recreation centres and parks are closed. Additionally, the lack of necessary equipment and professional guidance are also impediments to the practice of physical activity at home^(39)^.

Physical activity can play an important role in immune function, reducing the risk of developing and worsening chronic non-communicable diseases and obesity – risk factors for SARS-CoV-2 infection^(6,40)^. Considering this, WHO has launched a guide with tips on how to include physical activity in the daily routine^(41)^. The guide provides exercise suggestions with reference images and reinforces the recommendation that individuals practice at least 150–300 min/week of light/moderate physical activity or 75–150 min/week of vigorous physical activity^(42)^. In addition to these benefits, physical activity can also interfere with eating habits^(43)^ and, in our sample, the higher consumption of bakery products (OR = 1·104), meats (OR = 1·053), sweetened drinks (OR = 1·043) and the lower consumption of fresh fruits (OR = 0·858) were factors independently associated with not practicing physical activity.

An effort has also been made to encourage better eating habits in the quarantine period^(44)^, and in this study, some changes in eating habits have been noticeable. It was observed that some people stopped having breakfast, morning snack and lunch, while they increased the performance of evening snacks and additional meals. Although there is no evidence regarding the adequate number of meals, there is a discussion that the distribution of energy and nutrients between 4 and 5 meals can have a positive effect on health, since the fractionation of meals brings relief from digestive and metabolic overload caused by higher energy density meals, in addition to contributing to the fulfillment of the recommendations of the food and nutrient groups^(45)^. In our sample, people who reported consuming breakfast daily were less likely to smoke (OR = 0·256), as were those who had an afternoon snack (OR = 0·520). In addition, having breakfast was also inversely associated with the last quartile of screen time (OR = 0·596), while the consumption of afternoon snacks was more usual in those volunteers who practice some minutes of physical activity (OR = 0·632) and in those who sleep more than 7 h a night (OR = 0·591; *P* = 0·001).

Besides the reported changes in the number of meals, our volunteers also showed an increase in hours of sleep during the pandemic, and men were more likely to sleep 7 h or less (OR = 1·632). Sleeping more can justify the reduction in daytime meals and an increase in night time meals; on the other hand, meals close to bedtime can cause nighttime awakenings and worsen sleep quality and routine^(46)^. In our sample, consuming an evening snack was a factor independently associated with the first quartile of sleep time (OR = 1·505; *P* = 0·004).

Although only 3·9 % of volunteers related to skipping breakfast during the pandemic period, meta-analysis studies have shown that skipping breakfast is associated with a significantly increased risk of heart disease and overweight and obesity^(47,48)^. Breakfast skippers also had significantly worse indicators of quality of life than those who ate that meal, worse quality of the diet in general and worse perceptions of general health, social functioning, emotional role and mental health^(49,50)^. The decrease in the consumption of morning snacks and lunch was also observed by a small percentage of the volunteers. These habits have been associated with the increase in the frequency of evening snacks that can induce worse food choices, being associated with a lower inclusion of fruits and vegetables in the diet and outcomes of higher BMI, obesogenic dietary index and a higher percentage of time eating absently^(51,52)^.

Regarding the food choices reported by the volunteers, the results of the FFQ were very consistent in showing a worsening of the eating pattern, in which there is a decrease in consumption of fruits and vegetables and an increase in the consumption of candies and fast-food. Fruits and vegetables are rich sources of nutrients and bioactive compounds^(53)^, while candies and fast food are usually composed of ultra-processed and energy-dense foods with a high content of sugar, saturated and trans fats and poor in most micronutrients, fibres and proteins, and it is associated with greater risks of chronic diseases with an increased risk of overweight/obesity and metabolic syndrome^(54,55)^.

Negative changes in the food consumption profile were found in studies carried out with Brazilians^(11,12)^ and other populations during the quarantine period^(29,56,57)^. These changes also include low consumption of fruits and vegetables associated with increased consumption of sweets, and high consumption of snacks rich in energies, with low nutritional value^(5,9)^. Such findings have branded a global concern that has highlighted the need to create strategies that contribute to individuals’ health and well-being and the maintenance of healthy habits that can be harmed by security measures adopted to face the pandemic^(4–8,18,19,27,39,58)^.

These results bring a perspective and a base on the changes in the habits of the Brazilian population and agree with much of what has been observed in other populations^(59)^. In the literature, in addition to the changes observed in the perspective of worsening lifestyle habits^(59)^ – as a worsening of the eating pattern, increased consumption of alcoholic beverages, increased sedentary lifestyle – attention has grown over the consequences of the pandemic on psychological and mental health aspects^(17,60–62)^.

Although this study is on a high number of Brazilian individuals during quarantine outbreak pandemic for COVID-19, it has some limitations that deserve a discussion. The main limitation of this study is the use of a self-reported online questionnaire, which can lead to incorrect data filling and allow the participation of only people with internet access. Also, people were asked about a time before the pandemic, and very specific life/dietary issues, and some of them could not remember, or their answers may only reflect their impressions and notions on how the pandemic is affecting them. A strength of our study is the application of the questionnaire 5 months after the start of the pandemic, period of high adhesion of restrictive measures of social isolation in Brazil, being possible to notice the changes that occurred during this period.

In conclusion, the isolation measures adopted in Brazil caused changes in the daily lives of individuals, reflecting an increase in hours worked, screen time, hours of sleep, smoking and drinking frequency. On the other hand, there was a reduction in the dose of alcoholic beverages but also in the practice of physical activity. Eating habits also changed, reducing the performance of daytime meals and increasing the performance of nighttime meals. The frequency of consumption of instant meals and fast food has increased, while consumption of fruits and vegetables has decreased. Studying the repercussions of the pandemic on all these aspects is extremely important. Future studies should deepen this theme to support creating and implementing appropriate health promotion strategies in the current public health emergency.

## Supplementary material

For supplementary material accompanying this paper visit http://dx.doi.org/10.1017/S136898002100255X. click here to view supplementary material
