# Supplementary material for: Lifestyle and eating habits before and during COVID-19 quarantine in Brazil
Source: Public Health Nutr. 2021 Jun 10;25(1):65–75. doi: 10.1017/S136898002100255X (PMC8280396; doi:10.1017/S136898002100255X)
Supplement: Supplementary file 1 [file S136898002100255Xsup.zip › S136898002100255Xsup001.docx]

**Supplementary Table 1. Variables associated with the frequency of alcoholic beverage consumption during the pandemic period in Brazil by univariate logistic regression analyses**

| Variables | UNIVARIATE | | |
| --- | --- | --- | --- |
|  | **OR** | **95%CI** | **P value** |
| Socioeconomic data | | | |
| Gender (female) | 1.093 | 0.834 - 1.433 | 0.519 |
| Age (years) | 1.027 | 1.017 - 1.036 | <0.001 |
| Per capita income (R$) | 1.000 | 1.000 - 1.000 | <0.001 |
| Number of people living together | 0.901 | 0.823 - 0.986 | 0.023 |
| Living with children | 1.757 | 1.373 - 2.248 | <0.001 |
| Living with parents | 0.495 | 0.393 - 0.624 | <0.001 |
| Social/physical distancing | 0.968 | 0.778 - 1.204 | 0.770 |
| Education level | | | |
| Post-graduate | 2.011 | 1.615 - 2.505 | <0.001 |
| Graduate | 0.528 | 0.424 - 0.658 | <0.001 |
| College or less | 0.744 | 0.458 - 1.208 | 0.232 |
| Labor situation during pandemic | | | |
| Remotely full/part-time work or study | 0.810 | 0.640 - 1.025 | 0.080 |
| Working or studying without alterations | 2.047 | 1.453 - 2.883 | <0.001 |
| Increase in time spent at work (including household chores) | 0.764 | 0.607 - 0.962 | 0.022 |
| Lifestyle data | | | |
| Screen time during pandemic (hours/day) | 0.998 | 0.971 - 1.026 | 0.884 |
| Dose of alcoholic beverage during pandemic (dose per occasion) | 2.598 | 2.338 - 2.888 | <0.001 |
| Smoking during pandemic (cigarettes/day) | 1.067 | 1.027 - 1.107 | 0.001 |
| Sleep time during pandemic (hours/day) | 1.043 | 0.961 - 1.131 | 0.316 |
| Physical activity during pandemic (minutes/week) | 1.001 | 1.000 - 1.003 | 0.025 |
| Dietary consumption and eating habits during the pandemic | | | |
| Daily meals |  |  |  |
| Breakfast | 1.138 | 0.844 - 1.536 | 0.397 |
| Morning snack | 1.007 | 0.801 - 1.267 | 0.949 |
| Lunch | 2.216 | 1.041 - 4.719 | 0.039 |
| Afternoon snack | 0.903 | 0.688 - 1.185 | 0.463 |
| Dinner | 1.304 | 0.981 - 1.732 | 0.067 |
| Evening snack | 0.900 | 0.708 - 1.143 | 0.388 |
| Other meals during pandemic | 0.827 | 0.586 - 1.168 | 0.281 |
| Number of meals | 1.017 | 0.923 - 1.120 | 0.738 |
| Frequency of weekly consumption |  |  |  |
| Legumes | 0.958 | 0.926 - 0.991 | 0.014 |
| Cereals | 0.958 | 0.922 - 0.996 | 0.031 |
| Bakery products | 0.997 | 0.965 - 1.031 | 0.866 |
| Milk and dairy | 0.978 | 0.948 - 1.010 | 0.176 |
| Fresh fruits | 0.989 | 0.959 - 1.019 | 0.465 |
| Meat | 1.011 | 0.977 - 1.046 | 0.543 |
| Hamburgers and canned products | 0.988 | 0.948 - 1.029 | 0.561 |
| Vegetables | 1.019 | 0.985 - 1.055 | 0.280 |
| Sweetened drinks | 0.967 | 0.935 - 1.001 | 0.055 |
| Instant meals and snacks | 0.955 | 0.913 - 0.998 | 0.040 |
| Candies | 1.016 | 0.983 - 1.049 | 0.347 |
| Fast-food | 1.008 | 0.963 - 1.055 | 0.728 |

**Supplementary Table 2. Variables associated with dose of alcoholic beverage consumption during the pandemic period in Brazil by univariate logistic regression analyses**

| Variables | UNIVARIATE | | |
| --- | --- | --- | --- |
|  | **OR** | **95%CI** | **P value** |
| Socioeconomic data | | | |
| Gender (male) | 1.656 | 1.197 - 2.045 | 0.001 |
| Age (years) | 1.005 | 0.996 - 1.014 | 0.316 |
| Per capita income (R$) | 1.000 | 1.000 - 1.000 | 0.144 |
| Number of people living together | 0.954 | 0.873 - 1.042 | 0.294 |
| Living with children | 1.123 | 0.879 - 1.436 | 0.354 |
| Living with parents | 0.748 | 0.599 - 0.934 | 0.010 |
| Social/physical distancing | 1.246 | 0.669 - 2.320 | 0.489 |
| Education level | | | |
| Post-graduate | 1.119 | 0.903 - 1.386 | 0.305 |
| Graduate | 0.943 | 0.762 - 1.169 | 0.594 |
| College or less | 0.774 | 0.482 - 1.243 | 0.289 |
| Labor situation during pandemic | | | |
| Remotely full/part-time work or study | 0.872 | 0.690 - 1.102 | 0.251 |
| Working or studying without alterations | 1.708 | 1.2014 - 2.403 | 0.002 |
| Increase in time spent at work (including household chores) | 1.018 | 0.812 - 1.275 | 0.880 |
| Lifestyle data | | | |
| Screen time during pandemic (hours/day) | 1.006 | 0.979 - 1.034 | 0.653 |
| Frequency of alcoholic beverage during pandemic (times/week) | 3.769 | 3.193 - 4.449 | <0.001 |
| Smoking during pandemic (cigarettes/day) | 1.112 | 1.062 - 1.165 | <0.001 |
| Sleep time during pandemic (hours/day) | 1.051 | 0.970 - 1.140 | 0.223 |
| Physical activity during pandemic (minutes/week) | 1.002 | 1.001 - 1.003 | 0.001 |
| Dietary consumption and eating habits during the pandemic | | | |
| Daily meals |  |  |  |
| Breakfast | 0.724 | 0.541 - 0.970 | 0.030 |
| Morning snack | 0.874 | 0.695 - 1.097 | 0.245 |
| Lunch | 1.922 | 0.945 - 3.906 | 0.071 |
| Afternoon snack | 0.897 | 0.685 - 1.174 | 0.428 |
| Dinner | 1.246 | 0.943 - 1.647 | 0.121 |
| Evening snack | 1.017 | 0.804 - 1.288 | 0.885 |
| Other meals during pandemic | 1.114 | 0.798 - 1.556 | 0.525 |
| Number of meals | 0.979 | 0.890 -1.078 | 0.668 |
| Frequency of weekly consumption |  |  |  |
| Legumes | 0.961 | 0.929 - 0.995 | 0.023 |
| Cereals | 0.963 | 0.927 - 1.001 | 0.055 |
| Bakery products | 0.953 | 0.922 - 0.985 | 0.004 |
| Milk and dairy | 0.959 | 0.929 - 0.989 | 0.009 |
| Fresh fruits | 0.954 | 0.926 - 0.983 | 0.002 |
| Meat | 0.994 | 0.961 - 1.028 | 0.711 |
| Hamburgers and canned products | 0.997 | 0.958 - 1.039 | 0.899 |
| Vegetables | 0.988 | 0.955 - 1.022 | 0.488 |
| Sweetened drinks | 0.993 | 0.961 - 1.026 | 0.668 |
| Instant meals and snacks | 0.964 | 0.923 - 1.007 | 0.097 |
| Candies | 0.976 | 0.945 - 1.008 | 0.136 |
| Fast-food | 0.996 | 0.952 - 1.043 | 0.874 |

**Supplementary Table 3. Variables associated with the smoking habit during the pandemic period in Brazil by univariate logistic regression analyses**

| Variables | UNIVARIATE | | |
| --- | --- | --- | --- |
|  | **OR** | **95%CI** | **P value** |
| Socioeconomic data | | | |
| Gender (male) | 1.453 | 0.925 – 2.283 | 0.105 |
| Age (years) | 1.045 | 1.030 – 1.060 | <0.001 |
| Per capita income (R$) | 1.000 | 1.000 – 1.000 | 0.069 |
| Number of people living together | 0.988 | 0.841 – 1.162 | 0.886 |
| Living with children | 0.526 | 0.349 – 0.791 | 0.002 |
| Living with parents | 1.619 | 1.052 – 2.493 | 0.028 |
| Social/physical distancing | 0.936 | 0.629 – 1.393 | 0.745 |
| Education level | | | |
| Post-graduate | 0.645 | 0.430 – 0.967 | 0.034 |
| Graduate | 1.033 | 0.698 – 1.528 | 0.871 |
| College or less | 3.379 | 1.874 – 6.093 | <0.001 |
| Labor situation during pandemic | | | |
| Remotely full/part-time work or study | 0.561 | 0.376 – 0.837 | 0.005 |
| Working or studying without alterations | 0.623 | 0.297 – 1.307 | 0.211 |
| Increase in time spent at work (including household chores) | 1.039 | 0.689 – 1.567 | 0.855 |
| Lifestyle data | | | |
| Screen time during pandemic (hours/day) | 1.018 | 0.968 – 1.070 | 0.494 |
| Frequency of alcoholic beverage during pandemic (times/week) | 1.444 | 1.276 – 1.634 | <0.001 |
| Dose of alcoholic beverage during pandemic (dose per occasion) | 1.304 | 1.185 – 1.435 | <0.001 |
| Sleep time during pandemic (hours/day) | 0.910 | 0.785 – 1.054 | 0.209 |
| Physical activity during pandemic (minutes/week) | 0.999 | 0.997 – 1.002 | 0.473 |
| Dietary consumption and eating habits during the pandemic | | | |
| Daily meals |  |  |  |
| Breakfast | 0.243 | 0.160 – 0.368 | <0.001 |
| Morning snack | 0.867 | 0.567 – 1.327 | 0.512 |
| Lunch | 0.092 | 0.047 – 0.180 | <0.001 |
| Afternoon snack | 0.343 | 0.227 – 0.518 | <0.001 |
| Dinner | 0.512 | 0.331 – 0.793 | <0.001 |
| Evening snack | 0.840 | 0.538 – 1.313 | 0.445 |
| Other meals during pandemic | 1.150 | 0.639 – 2.070 | 0.641 |
| Number of meals | 0.561 | 0.474 – 0.664 | <0.001 |
| Frequency of weekly consumption |  |  |  |
| Legumes | 0.976 | 0.918 – 1.037 | 0.433 |
| Cereals | 0.955 | 0.893 – 1.022 | 0.186 |
| Bakery products | 0.995 | 0.937 – 1.056 | 0.871 |
| Milk and dairy | 0.964 | 0.911 – 1.020 | 0.204 |
| Fresh fruits | 0.915 | 0.866 – 0.966 | 0.001 |
| Meat | 0.995 | 0.936 – 1.058 | 0.878 |
| Hamburgers and canned products | 1.163 | 1.095 – 1.236 | <0.001 |
| Vegetables | 0.951 | 0.895 – 1.010 | 0.103 |
| Sweetened drinks | 1.121 | 1.064 – 1.181 | <0.001 |
| Instant meals and snacks | 1.156 | 1.087 – 1.229 | <0.001 |
| Candies | 1.016 | 0.959 – 1.077 | 0.587 |
| Fast-food | 1.148 | 1.073 – 1.228 | <0.001 |

**Supplementary Table 4. Variables associated with the screen time during the pandemic period in Brazil by univariate logistic regression analyses**

| Variables | UNIVARIATE | | |
| --- | --- | --- | --- |
|  | **OR** | **95%CI** | **P value** |
| Socioeconomic data | | | |
| Gender (male) | 1.010 | 0.774 - 1.319 | 0.939 |
| Age (years) | 0.965 | 0.955 - 0.974 | <0.001 |
| Per capita income (R$) | 1.000 | 1.000 - 1.000 | 0.489 |
| Number of people living together | 1.044 | 0.957 - 1.140 | 0.332 |
| Living with children | 0.527 | 0.410 - 0.677 | <0.001 |
| Living with parents | 1.805 | 1.448 - 2.250 | <0.001 |
| Social/physical distancing | 1.120 | 0.904 - 1.388 | 0.300 |
| Education level | | | |
| Post-graduate | 0.916 | 0.740 - 1.133 | 0.417 |
| Graduate | 1.253 | 1.013 - 1.549 | 0.038 |
| College or less | 0.512 | 0.315 - 0.832 | 0.007 |
| Labor situation during pandemic | | | |
| Remotely full/part-time work or study | 2.678 | 2.096 - 3.421 | <0.001 |
| Working or studying without alterations | 0.327 | 0.223 - 0.479 | <0.001 |
| Increase in time spent at work (including household chores) | 0.544 | 0.433 - 0.683 | <0.001 |
| Lifestyle data | | | |
| Frequency of alcoholic beverage during pandemic (times/week) | 1.013 | 0.936 - 1.097 | 0.746 |
| Dose of alcoholic beverage during pandemic (dose per occasion) | 1.020 | 0.963 - 1.081 | 0.492 |
| Smoking during pandemic (cigarettes/day) | 1.017 | 0.983 - 1.053 | 0.325 |
| Sleep time during pandemic (hours/day) | 0.860 | 0.793 - 0.933 | <0.001 |
| Physical activity during pandemic (minutes/week) | 0.999 | 0.998 - 1.000 | 0.084 |
| Dietary consumption and eating habits during the pandemic | | | |
| Daily meals |  |  |  |
| Breakfast | 0.617 | 0.459 - 0.829 | 0.001 |
| Morning snack | 0.989 | 0.789 - 1.238 | 0.921 |
| Lunch | 0.771 | 0.403 - 1.475 | 0.433 |
| Afternoon snack | 1.015 | 0.776 - 1.327 | 0.914 |
| Dinner | 1.213 | 0.922 - 1.595 | 0.168 |
| Evening snack | 1.230 | 0.973 - 1.555 | 0.083 |
| Other meals during pandemic | 1.063 | 0.762 - 1.482 | 0.721 |
| Number of meals | 1.006 | 0.914 - 1.106 | 0.907 |
| Frequency of weekly consumption |  |  |  |
| Legumes | 0.995 | 0.962 - 1.029 | 0.749 |
| Cereals | 1.001 | 0.964 - 1.040 | 0.952 |
| Bakery products | 0.994 | 0.962 - 1.026 | 0.696 |
| Milk and dairy | 0.998 | 0.967 - 1.030 | 0.905 |
| Fresh fruits | 0.965 | 0.937 - 0.995 | 0.022 |
| Meat | 0.990 | 0.957 - 1.023 | 0.540 |
| Hamburgers and canned products | 1.010 | 0.970 - 1.051 | 0.643 |
| Vegetables | 0.952 | 0.921 - 0.986 | 0.005 |
| Sweetened drinks | 1.018 | 0.985 - 1.052 | 0.292 |
| Instant meals and snacks | 1.015 | 0.973 - 1.058 | 0.494 |
| Candies | 1.026 | 0.994 - 1.059 | 0.115 |
| Fast-food | 0.995 | 0.951 - 1.041 | 0.838 |

**Supplementary Table 5. Variables associated with the frequency of physical activity during the pandemic period in Brazil by univariate logistic regression analyses**

| Variables | UNIVARIATE | | |
| --- | --- | --- | --- |
|  | **OR** | **95%CI** | **P value** |
| Socioeconomic data | | | |
| Gender (female) | 1.337 | 1.008 - 1.773 | 0.044 |
| Age (years) | 1.009 | 0.999 - 1.018 | 0.068 |
| Per capita income (R$) | 1.000 | 1.000 - 1.000 | 0.010 |
| Number of people living together | 0.999 | 0.913 - 1.093 | 0.985 |
| Living with children | 1.498 | 1.169 - 1.919 | 0.001 |
| Living with parents | 0.836 | 0.667 - 1.048 | 0.120 |
| Social/physical distancing | 0.965 | 0.774 - 1.204 | 0.753 |
| Education level | | | |
| Post-graduate | 0.903 | 0.725 - 1.124 | 0.359 |
| Graduate | 0.994 | 0.799 - 1.273 | 0.960 |
| College or less | 1.630 | 1.028 - 2.584 | 0.038 |
| Labor situation during pandemic | | | |
| Remotely full/part-time work or study | 0.650 | 0.513 - 823 | <0.001 |
| Working or studying without alterations | 1.444 | 1.026 - 2.031 | 0.035 |
| Increase in time spent at work (including household chores) | 0.914 | 0.726 - 1.152 | 0.447 |
| Lifestyle data | | | |
| Screen time during pandemic (hours/day) | 1.021 | 0.993 - 1.050 | 0.138 |
| Frequency of alcoholic beverage during pandemic (times/week) | 0.915 | 0.841 - 0.995 | 0.039 |
| Dose of alcoholic beverage during pandemic (dose per occasion) | 0.880 | 0.827 - 0.936 | <0.001 |
| Smoking during pandemic (cigarettes/day) | 1.016 | 0.982 - 1.051 | 0.361 |
| Sleep time during pandemic (hours/day) | 0.895 | 0.824 - 0.972 | 0.009 |
| Dietary consumption and eating habits during the pandemic | | | |
| Daily meals |  |  |  |
| Breakfast | 1.070 | 0.792 - 1.447 | 0.659 |
| Morning snack | 0.780 | 0.617 - 0.987 | 0.038 |
| Lunch | 1.343 | 0.672 - 2.686 | 0.404 |
| Afternoon snack | 0.677 | 0.516 - 0.888 | 0.005 |
| Dinner | 0.822 | 0.623 - 1.085 | 0.167 |
| Evening snack | 0.935 | 0.734 - 1.191 | 0.586 |
| Other meals during pandemic | 1.663 | 1.1991 - 2.322 | 0.003 |
| Number of meals | 0.930 | 0.843 - 1.026 | 0.146 |
| Frequency of weekly consumption |  |  |  |
| Legumes | 1.003 | 0.969 - 1.038 | 0.868 |
| Cereals | 1.016 | 0.977 - 1.057 | 0.421 |
| Bakery products | 1.082 | 1.045 - 1.120 | <0.001 |
| Milk and dairy | 0.987 | 0.956 - 1.019 | 0.413 |
| Fresh fruits | 0.896 | 0.869 - 0.925 | <0.001 |
| Meat | 1.049 | 1.013 - 1.087 | 0.007 |
| Hamburgers and canned products | 1.092 | 1.048 - 1.137 | <0.001 |
| Vegetables | 0.934 | 0.902 - 0.967 | <0.001 |
| Sweetened drinks | 1.083 | 1.048 - 1.120 | <0.001 |
| Instant meals and snacks | 1.070 | 1.026 - 1.117 | 0.002 |
| Candies | 1.053 | 1.019 - 1.088 | 0.002 |
| Fast-food | 1.027 | 0.981 - 1.075 | 0.262 |
|  | | | |

**Supplementary Table 6. Variables associated with the sleep time during the pandemic period in Brazil by univariate logistic regression analyses**

| Variables | UNIVARIATE | | |
| --- | --- | --- | --- |
|  | **OR** | **95%CI** | **P value** |
| Socioeconomic data | | | |
| Gender (male) | 1.536 | 1.149 - 2.053 | 0.004 |
| Age (years) | 1.000 | 0.990 - 1.011 | 0.948 |
| Per capita income (R$) | 1.000 | 1.000 - 1.000 | 0.043 |
| Number of people living together | 1.077 | 0.974 - 1.190 | 0.147 |
| Living with children | 1.225 | 0.932 - 1.610 | 0.145 |
| Living with parents | 0.997 | 0.777 - 1.279 | 0.978 |
| Social/physical distancing | 0.970 | 0.759 - 1.239 | 0.807 |
| Education level | | | |
| Post-graduate | 0.804 | 0.630 - 1.027 | 0.081 |
| Graduate | 1.337 | 1.049 - 1.704 | 0.019 |
| College or less | 0.982 | 0.383 - 1.215 | 0.194 |
| Labor situation during pandemic | | | |
| Remotely full/part-time work or study | 1.218 | 0.928 - 1.599 | 0.155 |
| Working or studying without alterations | 1.177 | 0.808 - 1.715 | 0.396 |
| Increase in time spent at work (including household chores) | 0.528 | 0.401 - 0.965 | <0.001 |
| Lifestyle data | | | |
| Screen time during pandemic (hours/day) | 1.065 | 1.033 - 1.098 | <0.001 |
| Frequency of alcoholic beverage during pandemic (times/week) | 0.923 | 0.839 - 1.016 | 0.103 |
| Dose of alcoholic beverage during pandemic (dose per occasion) | 0.984 | 0.921 - 1.052 | 0.639 |
| Smoking during pandemic (cigarettes/day) | 1.021 | 0.985 - 1.059 | 0.251 |
| Physical activity during pandemic (minutes/week) | 0.999 | 0.997 - 1.000 | 0.042 |
| Dietary consumption and eating habits during the pandemic | | | |
| Daily meals |  |  |  |
| Breakfast | 1.176 | 0.835 - 1.657 | 0.353 |
| Morning snack | 1.019 | 0.788 - 1.318 | 0.884 |
| Lunch | 1.124 | 0.527 - 2.399 | 0.762 |
| Afternoon snack | 0.622 | 0.466 - 0.832 | 0.001 |
| Dinner | 0.847 | 0.625 - 1.149 | 0.286 |
| Evening snack | 1.539 | 1.189 - 1.993 | 0.001 |
| Other meals during pandemic | 1.599 | 1.122 - 2.279 | 0.009 |
| Number of meals | 1.056 | 0.947 - 1.178 | 0.329 |
| Frequency of weekly consumption |  |  |  |
| Legumes | 1.017 | 0.979 - 1.058 | 0.387 |
| Cereals | 0.976 | 0.935 - 1.019 | 0.265 |
| Bakery products | 0.981 | 0.945 - 1.017 | 0.296 |
| Milk and dairy | 0.978 | 0.943 - 1.013 | 0.208 |
| Fresh fruits | 0.968 | 0.935 - 1.001 | 0.059 |
| Meat | 0.985 | 0.949 - 1.023 | 0.441 |
| Hamburgers and canned products | 1.038 | 0.993 - 1.085 | 0.102 |
| Vegetables | 0.971 | 0.934 - 1.009 | 0.131 |
| Sweetened drinks | 1.031 | 0.995 - 1.070 | 0.094 |
| Instant meals and snacks | 1.015 | 0.968 - 1.064 | 0.534 |
| Candies | 0.991 | 0.956 - 1.028 | 0.642 |
| Fast-food | 0.985 | 0.935 - 1.038 | 0.574 |
